# Primary care provider referral patterns and awareness of biologic therapy for uncontrolled asthma

**DOI:** 10.1016/j.jacig.2025.100607

**Published:** 2025-11-17

**Authors:** Juan Carlos Cardet, Bijalben Patel, Phuong Bradford, Elisabeth Callen, Tarin L. Clay, Brenda Flam, John Fowler, Lucy Guerra, Christina Hester, R. Meredith Plant, Panida Sriaroon, Krizia Trasmonte, Dennis Ledford

**Affiliations:** a Division of Allergy and Immunology, Department of Internal Medicine, University of South Florida, Morsani College of Medicine, Tampa, Fla; b Division of Allergy and Immunology, Department of Internal Medicine, Baylor College of Medicine, Houston, Tex; c Department of Internal Medicine, University of South Florida, Morsani College of Medicine, Tampa, Fla; d DARTNet Institute, Aurora, Colo; e University of South Florida, Morsani College of Medicine, Tampa, Fla; f Division of General Internal Medicine, University of Florida, UF Health Shands Hospital, Gainesville, Fla; g Department of Pediatrics, University of South Florida, Morsani College of Medicine, Tampa, Fla; h Division of Allergy and Immunology, Department of Pediatrics, University of South Florida, Morsani College of Medicine, Tampa, Fla; i Division of Allergy and Immunology, Department of Internal Medicine, James A. Haley Veterans’ Hospital, Tampa, Fla

**Keywords:** Eosinophils, biomarkers, specialist, corticosteroids, monoclonal antibodies, biologics, asthma treatments, inflammation, primary care provider, outcomes

## Abstract

**Background:**

Asthma is a chronic airway disease associated with substantial morbidity. Most patients with uncontrolled asthma in the United States are managed by primary care providers (PCPs) and not by asthma specialists who are frequently updated on asthma treatment advances, including biologics, which decrease asthma exacerbation rates.

**Objective:**

We sought to investigate PCP referral patterns to asthma specialists, their familiarity with asthma biologic therapies, and the use of laboratory tests in asthma management.

**Methods:**

This was a cross-sectional survey study administered to PCPs in 47 US states. Respondent characteristics were analyzed using descriptive statistics. Bivariate analyses were conducted to examine associations between respondent characteristics and outcomes, with variables significant at *P* < .05 included in multivariable models.

**Results:**

The survey was completed by 404 PCPs, of whom 51.4% referred patients with uncontrolled asthma to a specialist after ≥3 annual exacerbations, 32.8% were unfamiliar with asthma biologic therapy, and 72.1% did not routinely order laboratory tests to guide management. PCPs who manage patients with asthma more frequently were more likely to be familiar with asthma biologics (odds ratio = 1.74, 95% CI 1.42-1.88, *P* = .001) and more likely to use laboratory data to aid in management (OR = 5.41, 95% CI 2.72-10.77, *P* < .001).

**Conclusion:**

Most PCPs delay specialist referrals until patients experience ≥3 annual asthma exacerbations and do not use laboratory tests in asthma management, and many are unfamiliar with asthma biologics. Enhancing communication and education between PCPs and specialists on asthma therapies may help reduce asthma exacerbations.

Asthma is a chronic inflammatory airway disease that affects more than 30 million people in the United States.^1^ Uncontrolled and exacerbation-prone asthma negatively impacts the quality of life of patients and their caregivers^2^ and drives most asthma-related health care expenditures,^3^ which are projected to exceed $960 billion in the next 20 years in the United States alone.^4^ In an effort to reduce this disease burden, the Global Initiative for Asthma 2025 guidelines recommend that primary care providers (PCPs) refer patients to an asthma care specialist (typically an allergy/immunology specialist or pulmonologist) if they require 2 or more courses of oral corticosteroids in 1 year.^5^ Referrals to asthma care specialists can reduce asthma morbidity,^6^ partly because allergists and pulmonologists are better prepared to evaluate and treat complex asthma, including prescribing asthma biologics shown to reduce exacerbation rates.^7^, ^8^, ^9^, ^10^, ^11^ Currently, 6 biologics are approved by the US Food and Drug Administration for asthma treatment: omalizumab, mepolizumab, reslizumab, benralizumab, dupilumab, and tezepelumab. These biologics reduce asthma exacerbations by 50% or more, with post hoc analyses showing up to 70% exacerbation reduction in select subpopulations.^12^, ^13^, ^14^ Furthermore, these approved therapies for asthma are corticosteroid-sparing agents^15^ and can reduce the multiple, cumulative side effects associated with systemic and high-dose inhaled corticosteroids.^16^^,^^17^ However, biologics for asthma are underprescribed, even among eligible patients with uncontrolled disease.^18^, ^19^, ^20^

The reasons underlying limited uptake of biologics for asthma are multifactorial and include cost, requirement for parenteral administration, and logistical barriers to acquisition. In addition, PCPs manage more than 60% of patients with uncontrolled asthma, and more than half of patients do not visit an asthma care specialist after experiencing an exacerbation, which may limit awareness of new treatment options.^21^, ^22^, ^23^ In support of this, access to an asthma care specialist and insurance mandates are major predictors of being prescribed a biologic for asthma, as 91% of all asthma biologics are prescribed by specialists.^19^ This observation may be linked to patients preferring to receive biologics from asthma care specialists^24^ and asthma specialists being more aware of the potential benefits of biologic therapy.^25^, ^26^, ^27^

Little is known about reasons underlying referral patterns from PCPs to asthma care specialists. This may relate to unfamiliarity with indications for referral of uncontrolled or exacerbation-prone asthma or the perception that asthma is less severe because the disease responds to treatment with corticosteroids. Another possibility is that PCPs may delay referrals due to a lack of awareness that effective asthma biologics are safe treatment options. Finally, PCPs may not routinely use laboratory-based assessments to identify blood eosinophil counts (BECs) greater than 150 cells/μL as a trait treatable with asthma biologics.^28^^,^^29^ More specifically, PCPs may overlook BECs between 150 cells/μL and 500 cells/μL as the usual upper limit of normal is approximately 500 cells/μL. We hypothesized that PCPs delay in referring patients with uncontrolled asthma because of (1) lack of awareness of referral criteria for patients with uncontrolled and exacerbation-prone asthma to asthma care specialists, (2) limited knowledge of the existence and effectiveness of asthma biologics, and (3) lack of recognition that a BEC of >150 cells/μL identifies likely responders to biologics (ie, is a trait treatable with asthma biologics). To address these hypotheses, we conducted a survey-based study administered to a broad cross section of US primary care practices.

## Methods

This was a cross-sectional survey study administered to PCPs from 47 states and varied practice types. The survey was developed by our team of investigators comprising internists, family physicians, pediatricians, allied health staff, and allergists/immunologists. The survey was reviewed for internal consistency and clarity and to ensure that the questions were not leading. It was administered from December 1, 2024, to November 4, 2024 (Table E1 in this article’s Online Repository available at www.jaci-global.org). It was administered via e-mail and regular mail to approximately 11,000 physicians, nurse practitioners, and physician assistants who have consented for research participation; work in primary care; and are members of the American Academy of Family Physicians (AAFP) National Research Network, the AAFP, the American Medical Association, the University of South Florida Pediatrics Group, or the Tampa General Physicians’ Group. The survey could be completed online through a link with responses captured through Qualtrics (Provo, Utah) or through a paper version that was mailed to AAFP National Research Network members who indicated preference for paper surveys. Completion was voluntary, and each respondent was compensated with a $20 gift card. All duplicate records were deleted before data analysis.

Respondent characteristics captured with the survey included age, board certification type (eg, family medicine, internal medicine, nurse practitioner), practice size, practice zip code, frequency of managing patients with uncontrolled asthma, and experience with management of patients treated with asthma biologics. Rurality was determined by matching respondent zip codes with data available from Rural Health Research Centers using Categorization A, which approximates the urban, suburban (large rural city/town), and rural (small rural town/isolated small rural town) areas using zip codes.^30^ The US region was determined by using US Census Bureau categories.

Outcomes include assessment of percentage of patients with uncontrolled asthma referred to an asthma specialist, number of annual asthma exacerbations necessary to initiate a referral to an asthma care specialist, familiarity with asthma biologics and their initiation criteria, and frequency of use of routine blood tests to guide asthma management and their influence on the decision to refer to an asthma care specialists.

Respondent characteristics are presented using descriptive statistics. We tested the association between all respondent characteristics and each outcome in bivariate logistic regression analyses and selected the characteristics significant at *P* < .05 for inclusion in multivariable models. We report on predictive characteristics significant at *P* < .05 as odds ratios (ORs) for the most significant categories relative to referent groups (eg, relative to PCPs practicing in the Northeast, those practicing in the West were more likely to exhibit an outcome, with a given OR). We show in our figures the cohort distribution for the most important outcomes stratified by the most significant predictive characteristic identified in multivariable models. We used Stata SE version 17 (StataCorp, College Station, Texas) for all statistical analyses.

## Results

Of the 11,000 PCPs who were offered participation, 404 completed the survey (3.67%), and of those, 396 (98%) answered all questions. Physicians accounted for 92.5% of the responses, with the majority being board-certified in family medicine (86.5%), followed by internal medicine (3.8%) and pediatrics (2.2%) (Table I). More than two-thirds of respondents were age 40 years or older, more than 40% practiced in the South of the United States, and slightly more than one-third worked in practices with >20 clinicians. Nearly 60% of respondents manage patients with uncontrolled asthma once every 2 weeks or more frequently, and 60% do not comanage patients treated with an asthma biologic.

**Table I:** Characteristics of survey respondents (N = 404)

Characteristic	Respondents, no. (%)
Age (y)	
<40	132 (32.6)
40-59	191 (47.2)
≥60	82 (20.2)
Board certification	
Family medicine	347 (86.5)
Internal medicine	15 (3.8)
Pediatrics	9 (2.2)
Nurse practitioner	11 (2.8)
Physician assistant	10 (2.5)
Other	9 (2.2)
Practice size	
>20 clinicians	149 (36.8)
Practice location	
Urban	336 (84.6)
Suburban	30 (7.6)
Rural	31 (7.8)
US region	
Northeast	47 (11.8)
South	177 (44.6)
Midwest	102 (25.7)
West	71 (17.9)
Frequency managing patients with uncontrolled asthma	
Once per month or less	162 (40.7)
Sometimes, once every 2 weeks	179 (45.0)
At least once a week	57 (14.3)
Follows patients on asthma biologics	
No	239 (60)

Most PCPs (52.8%) refer 25% or fewer of their patients with uncontrolled asthma to asthma care specialists, which we describe as low-referring clinicians (LRCs). The main predictors of being an LRC were the US region of practice and the frequency of managing patients with uncontrolled asthma. Compared with PCPs practicing in urban areas, those in rural areas were more likely to be LRCs (OR = 5.76, 95% CI 1.95-16.98, *P* = .001). Compared with PCPs who manage patients with uncontrolled asthma once a month or less frequently, those who do so more frequently were more likely to be LRCs (OR = 1.42, 95% CI 1.05-1.91, *P* = .02).

**Fig 1:**
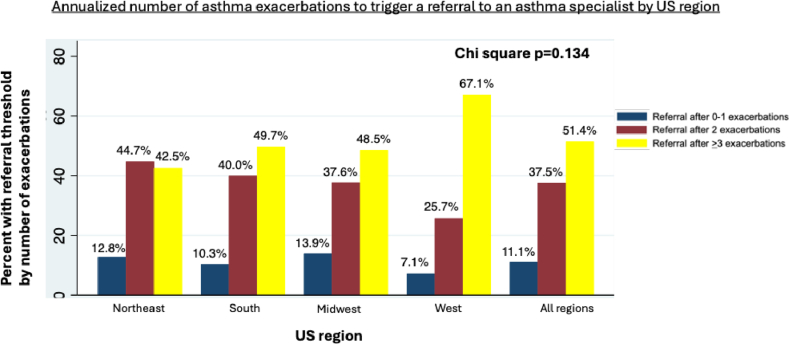
US region was the most significant predictor for delaying referrals of patients with uncontrolled asthma to specialists until after they experience ≥3 exacerbations. Compared with the Northeast, PCPs in the West were more likely to delay referrals (OR = 2.93, 95% CI 1.33-6.46, *P* = .008). Referral thresholds were compared across US regions using χ^2^.

More than half of PCPs (51.4%) refer patients with uncontrolled asthma to an asthma care specialist after they experience ≥3 exacerbations per year (Fig 1). The main predictors for referring patients with uncontrolled asthma after they experience ≥3 exacerbations per year were their board certification, US region of their practice, and frequency of managing patients with uncontrolled asthma. Compared with family medicine clinicians, internists were less likely to refer to asthma care specialists after ≥3 asthma exacerbations (OR = 0.24, 95% CI 0.07-0.91, *P* = .036). However, it is important to note that 86.6% of PCPs surveyed were family medicine clinicians, and only 3.8% were internal medicine clinicians. PCPs managing patients with uncontrolled asthma more frequently than once a month were more likely to refer patients to asthma care specialists only after they experience ≥3 exacerbations per year (OR = 1.41, 95% CI 1.03-1.93, *P* = .032). Finally, relative to PCPs practicing in the Northeast, those practicing in the West were more likely to refer to asthma care specialists only after ≥3 asthma exacerbations (OR = 2.93, 95% CI 1.33-6.46, *P* = .008).

**Fig 2:**
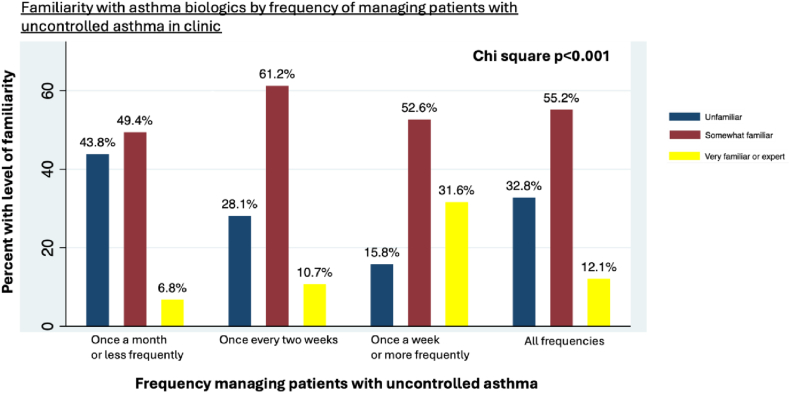
Frequency of managing patients with uncontrolled asthma was the most significant predictor for familiarity with asthma biologics. PCPs who saw such patients weekly or more often were likely to be familiar with biologics (OR = 0.26, 95% CI 0.12-0.58, *P* = .001) compared with PCPs seeing patients less frequently. Familiarity was compared across these management frequency categories using χ^2^.

We also evaluated familiarity of PCPs with asthma biologics and found that nearly one-third (32.8%) were unfamiliar with biologics and that almost half (48.6%) were unfamiliar with the criteria for biologic initiation. The key factors predicting familiarity with asthma biologics were the frequency of managing patients with uncontrolled asthma and experience with patients treated with asthma biologics. PCPs who managed patients with uncontrolled asthma once a week or more frequently were more likely to be familiar with asthma biologics compared with PCPs who managed them less often (OR = 1.74, 95% CI 1.42-1.88, *P* = .001). A similar pattern was observed for familiarity with biologic initiation criteria (Fig 2). Furthermore, clinicians who comanage patients treated with asthma biologics were more likely to be familiar with these biologics (OR = 1.70, 95% CI 1.51-1.82, *P* < .001), with similar results for familiarity with the initiation criteria. Additionally, respondents ages 40 years or older were more likely to be familiar with asthma biologic initiation criteria (OR = 1.29, 95% CI 1.05-1.47, *P* = .023) compared with their younger counterparts.

**Fig 3:**
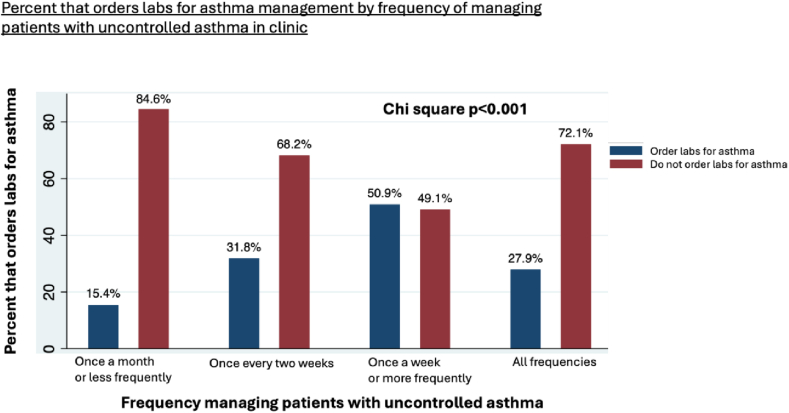
Frequency of managing patients with uncontrolled asthma was the strongest predictor for ordering laboratory tests. Clinicians managing patients weekly or more frequently were more likely to order laboratory tests (OR=5.41, 95% CI 2.72—10.77, p<0.001) compared with clinicians managing patients monthly or less frequently. Ordering laboratory tests was compared across these management frequency categories using χ^2^. *Labs*, Laboratory tests.

Laboratory values help identify treatable asthma traits, such as eosinophilic and allergic asthma phenotypes, which are linked to exacerbation risk and guide biologic eligibility. We found that nearly three-quarters of PCPs (72.1%) do not routinely order blood tests to guide asthma management, and fewer than 18% order BECs. Key predictors for routinely ordering laboratory tests in asthma management included older age of the clinician, experience treating patients with uncontrolled asthma, and managing patients on asthma biologics. Respondents ages 40 years and older and those comanaging patients on biologic therapy were more likely than their counterparts to order laboratory tests to guide asthma management (OR = 1.53, 95% CI 1.10-2.12, *P* = .011, and OR = 2.20, 95% CI 1.38-3.51, *P* = .001, respectively). Additionally, PCPs who managed patients with uncontrolled asthma once a week or more frequently were more likely to order laboratory tests than those who did so once a month or less (OR = 5.41, 95% CI 2.72-10.77, *P* < .001) (Fig 3). Finally, clinicians comanaging patients treated with asthma biologics were more likely to order laboratory tests compared with clinicians who did not manage such patients (OR = 2.20, 95% CI 1.38-3.51, *P* = .001).

## Discussion

To our knowledge, this is the first study to report that most PCPs do not refer patients with uncontrolled asthma until after they experience ≥3 exacerbations per year, that most do not utilize laboratory tests to guide asthma management, and that many are unfamiliar with asthma biologics, which reflects gaps in medical education and in communication between PCPs and asthma care specialists. Our study showed that more than half of PCPs refer 25% or fewer of their patients with uncontrolled asthma to asthma care specialists. This is consistent with prior findings showing that only 8% of patients with uncontrolled asthma were referred to specialists within 24 months after the initial diagnosis.^27^ We suspect that reduced referral patterns are more common in rural areas due to limited access to specialists. Another explanation is that PCPs who frequently manage patients with uncontrolled asthma may feel confident in managing these patients, which can also lead to fewer referrals. This further provides an opportunity to educate PCPs about the broader range of corticosteroid-sparing treatment options, which specialists are more likely to use than PCPs.

Our study also showed that most PCPs delay referrals to asthma care specialists until a patient has experienced ≥3 exacerbations in a year. Current guidelines recommend that patients with severe asthma who have ≥2 exacerbations annually should be referred to a specialist and considered for biologic therapies.^5^ Despite these recommendations, most patients who meet these criteria are not referred promptly, and there is a median delay of 880 days between eligibility and referral.^31^ Delaying referrals prevents timely access to advanced treatments, which can potentially lead to prolonged periods of uncontrolled asthma and increased morbidity. These patients are also at greater risk of increased corticosteroid use, which can result in significant negative outcomes, such as diabetes, osteoporosis, hypertension, accelerated atherosclerosis, increased risk of deep vein thrombosis, and mental health issues.^16^ Referring to a specialist even after a single exacerbation can help optimize asthma management and reduce reliance on systemic corticosteroids. These findings suggest the need for more discrete and actionable recommendations on specialist referrals in national asthma guidelines. Greater education about these guidelines is also needed among PCPs, including where and how to access them.

We also found that nearly one-third of PCPs are unfamiliar with asthma biologics, even though the first asthma biologic became available more than 20 years ago. This lack of familiarity may result from several factors, including limited interactions between PCPs and asthma care specialists, guidelines recommending initiating asthma biologics only after failure of other therapies, the availability of multiple asthma treatment options (ie, inhalers and oral medicines), the efficacy of systemic corticosteroids for most asthma cases, limited awareness of risk due to cumulative lifetime intermittent systemic corticosteroid and prolonged high-dose inhaled corticosteroid burden, and the limited education on asthma biologics among PCPs. Our results show that the primary predictor of unfamiliarity with asthma biologics is less frequent management of patients with uncontrolled asthma, as well as not comanaging patients treated with asthma biologics, suggesting that familiarity with asthma biologics comes with experience. These findings suggest the need for better asthma education in residency programs, considering that younger physicians were found to be less familiar with asthma biologic initiation criteria.

Another key goal in this study was to assess use by PCPs of blood tests such as BECs for asthma management. Eosinophils play a key role in asthma pathobiology, driving airway inflammation, mucus plugging, bronchoconstriction, and airway remodeling.^32^ BECs not only are useful as a prognostic factor for uncontrolled asthma, but also serve as a biomarker for responsiveness to inhaled corticosteroids and biologic therapies.^1^ Asthma exacerbation risk increases as BECs rise, but most patients with asthma remain below the upper limit of the normal range (approximately 500 eosinophils/μL) for most laboratories. We found that nearly three-fourths of PCPs do not use laboratory tests to assist in asthma management. Similar to unfamiliarity with asthma biologics, the main predictors for not utilizing laboratory tests included less frequent management of patients with uncontrolled asthma and not comanaging patients on asthma biologics. One reason for the limited use of BECs in asthma management may be that the threshold for greater risk of asthma exacerbations and for predicting benefit from asthma biologics falls in the low-normal range, ie, ≥150 eosinophils/μL, which many PCPs may therefore overlook. As complete blood count with differential results are available for most patients from the prior 2 to 3 years in PCP clinics, targeted educational efforts to increase PCP use of BECs in asthma management is feasible.

Our study has several limitations, many of which are inherent to its survey design. First, self-assessments of knowledge on topics such as asthma biologics using terms such as expert is subject to interindividual variability in interpretation. Regardless, the fact that nearly one-third of respondents assessed themselves to be unfamiliar with asthma biologics is worrisome. Second, we did not capture attendance of PCPs of continuing medical education programs on asthma management or their treatment algorithms for uncontrolled asthma, both of which were also likely variable across respondents. Again, the fact that most PCPs refer patients to asthma specialists only after they experience ≥3 exacerbations is concerning regardless of any deficiencies that might be observed in their continuing medical education or their management algorithms. Third, our survey response rate was low (3.67%), which limits the national representativeness of our results. However, our sample size (N = 404) was large enough and our survey was administered to a sufficiently broad cross-section of PCPs from 47 states and varied practice types to yield novel and valuable insights into asthma management in our current health care system. Fourth, 2 CIs for our ORs were wide (ie, PCPs practicing in rural areas having OR = 5.76, 95% CI 1.95-16.98, of being LRCs vs PCPs practicing in urban areas and internists having OR = 0.24, 95% CI 0.07-0.91, of referring to specialists after ≥3 exacerbations), which suggests possible imprecision in our estimates. Although predictor estimates may be imprecise, they do not detract from our primary finding—that most PCPs are delaying referrals of patients with uncontrolled asthma to specialty care. Fifth, the majority of the respondents were family medicine physicians, which reflects the specialty distribution of the convenience sample available to us. Our results might be different if pediatricians and internists were more equally represented. However, family medicine physicians account for a large proportion of all primary care visits in the United States, which highlights the relevance of our findings.^33^ Finally, we did not assess for other systematic barriers for PCPs to refer to asthma specialists in this survey (eg, unfamiliarity with specialists in their provider network, negative experiences with specialists in their system, a referral process that is overly cumbersome and time-consuming) that may underlie our results.

### Conclusion

Improving asthma outcomes requires a collaborative approach among both PCPs and asthma care specialists. Our study highlights the unmet need to identify optimal educational methods to reach, impact, and empower the PCP community to improve asthma care, as delays in shared decision-making discussions deprive patients of timely treatment options. Additionally, our study results can help refine targeted educational messages and, for example, discuss indications for referrals to asthma care specialists among PCPs who manage patients with uncontrolled asthma more frequently. Additional research into how PCPs prefer to receive updates on asthma management is needed to optimize uptake. In addition, a program that brings asthma specialists to PCP clinics, virtually or in person, for consultation on the management of patients with uncontrolled asthma might improve timely referral rates, in addition to the use of laboratory tests and biologics in asthma management. Such a program is currently being investigated in a US health care system. By improving knowledge and awareness of treatment options and treatable traits, PCPs will be better equipped to appropriately refer patients and optimize therapy.

### Declaration of generative AI and AI-assisted technologies in the writing process

During the preparation of this work the authors used ChatGPT-4 in order to improve the grammar and readability of the manuscript. After using this tool/service, the authors reviewed and edited the content and take full responsibility for the content of the publication.

## Disclosure statement

This study was conducted with funding from a GSK investigator-initiated award (to J.C.C.), in addition to an NHLBI R21 award (HL172124), the 10.13039/100001009Bristol Myers Squibb Foundation Winn Award, and the ALA/AAAAI Allergic Respiratory Diseases Award (AI-835475) (to J.C.C.).

Disclosure of potential conflict of interest: J.C. Cardet reports receiving honoraria from Aiolos-bio, Amgen, Apogee, AstraZeneca, Chiesi, GSK, Genentech, and Sanofi for work in advisory boards, steering committees, or giving educational lectures on asthma. D. Ledford reports receiving research support paid to his institution from AstraZeneca and Novartis, consultancy/advisory board honoraria for AstraZeneca and Sanofi/Regeneron, and honoraria for promotional and disease state awareness lectures from AstraZeneca/Amgen, GSK, Novartis, Genentech, and Sanofi/Regeneron. P. Sriaroon reports receiving honoraria from Genentech (advisory boards and Speakers Bureau). B. Flam reports stock investment in Pfizer. The rest of the authors declare that they have no relevant conflicts of interest.

## Supplementary data

Supplementary Table E1
