# Supplementary material for: Primary care provider referral patterns and awareness of biologic therapy for uncontrolled asthma
Source: J Allergy Clin Immunol Glob. 2025 Nov 17;5(2):100607. doi: 10.1016/j.jacig.2025.100607 (PMC12799774; doi:10.1016/j.jacig.2025.100607)
Supplement: Supplementary Table E1 [file mmc1.docx]

**Supplementary Table 1: The Survey**

1. **Primary board?**

- Family Medicine MD/DO
- Internal Medicine MD/DO
- Pediatrics MD/DO
- Nurse Practitioner
- Physician Assistant
- Nurse
- Psychologist
- Psychiatrist
- Other (please specify):

1. **Practice size: How many people work within your clinic in the following areas:**

MDs/DOs PAs/NPs

Other clinicians

Allied Health staff

Other staff

1. **What is your age?**

- Less than 25 years old
- 25-29 years
- 30-34 years
- 35-39 years
- 40-44 years
- 45-49 years
- 50-54 years
- 55-59 years
- 60-64 years
- 65-69 years
- 70-74 years
- 75-79 years
- 80 years or older

1. **What is the zip code of where you most commonly practice?**
2. **How often do you see patients with uncontrolled asthma in your clinic in a given one-month period? (Uncontrolled asthma is defined as patients who have symptoms more than twice per week, have nighttime symptoms, or have exacerbations requiring treatment with systemic corticosteroids).**

- Never
- Rarely, once per month
- Sometimes, once every two weeks
- Often, at least once a week
- Always, daily

1. **What percent of patients with uncontrolled asthma in your clinic do you refer to a pulmonologist or allergist?**

- 0%
- 1-25%
- 26-50%
- 51-75%
- 76-99%
- 100%

1. **After how many asthma exacerbations do you consider referring patients to a pulmonologist or allergist? An asthma exacerbation is defined as a deterioration in asthma symptoms requiring treatment with systemic corticosteroids or an emergency department or urgent care visit.**

- I refer patients to a pulmonologist or allergist prior to an exacerbation
- After 1 exacerbation in a year
- After 2 exacerbations in a year
- After more than 2 exacerbations in a year

1. **How would you rate your knowledge base regarding biologic therapy for asthma management?**

- Not familiar
- Somewhat familiar
- Very familiar
- Expert

1. **How would you rate your knowledge base regarding the criteria required for initiation of a biologic for asthma?**

- Not familiar
- Somewhat familiar
- Very familiar
- Expert

1. **Do you routinely manage patients on biologic therapy for asthma in your clinic?**

- Yes, I prescribe biologic therapy
- Yes, I have patients on biologic therapy prescribed by another provider
- No

1. **Do you routinely get blood tests to help guide asthma management in patients with uncontrolled asthma?**

- Yes
- No

1. **Which of the following? (**Display Question #12 if the answer to Question #11 was ‘yes’) **(Select all that apply):**

- CBC with differential
- IgE levels
- Allergen specific testing
- Total absolute eosinophil count

1. **Do lab results influence your therapeutic decision making for asthma for referral to a pulmonologist or allergist?**

- Yes
- No

**Please enter your email address to receive your e-gift card:**
